# Supplementary material for: Used Nasogastric Feeding Tubes from Neonates Contain Infant-Specific Bacterial Profiles
Source: Microorganisms. 2023 May 23;11(6):1365. doi: 10.3390/microorganisms11061365 (PMC10301528; doi:10.3390/microorganisms11061365)
Supplement: Supplementary file 1 [file microorganisms-11-01365-s001.zip › microorganisms-2305635-supplementary.pdf]

Supplementary Table S1. Clinical data of the infants (n = 34) and data regarding nasogastric feeding tubes (n = 94)

**Metadata at birth**	
Birth weight (g), median (IQR)	1083 (1259)
Gestational age (wks), median (IQR)	30.1 (7.6)
Male sex	20 (58.8%)
Cesarian section	22 (64.7%)
Multiple birth (mother)	14 (41.2%)
**While the tube was in place**	
Postnatal age (d), median (IQR)	37 (39)
Unpasteurized maternal milk feeding	85 (90.4%)
Probiotics (*Lactobacillus rhamnosus* GG and *Bifidobacterium animalis* ssp. *lactis)*	72 (76.6%)
Antibiotics	42 (44.7%)
Proton pump inhibitor	1 (1.1%)
Ventilation	
Mechanical	8 (8.5%)
Nasal c-pap	57 (60.6%)
Oxygen mask	3 (3.2%)
None	26 (27.7%)
Parenteral nutrition	21 (22.3%)
**Major outcomes**	
Sepsis (Positive blood culture and clinical signs of sepsis)	5 (14.7%)
Necrotizing enterocolitis (Bells modified stage II or III)	6 (17.6%)
Deceased	4 (11.8%)
**Number of tubes per infant**	
Number of tubes	Number of infants (%)
1	14 (41.2%)
2	8 (23.5%)
3	2 (5.9%)
4	3 (8.8%)
5	2 (5.9%))
6	1 (2.9%)
7	3 (8.8%)
9	1 (2.9%)

Supplementary Table S2. Table listing bacterial colonies identified with WGS across NG-tubes collected from eight preterm infants

	Child A	Child B	Child C	Child D	Child E	Child F	Child G	Child H
Klebsiella oxytoca	6/7 NG-tubes	6/7 NG-tubes	-	-	-	-	-	-
Enterobacter cloacae	-	-	2/4 NG tubes	7/9 NG-tubes	7/7 NG-tubes	-	-	-
Enterococcus faecium	-	-	3/4 NG-tubes	-	-	-	3/4 NG tubes	5/5 NG-tubes
Staphylococcus epidermidis	-	-	-	-	-	4/6 NG tubes	4/4 NG tubes	-
